# Synthetic Data-Driven Approaches for Chinese Medical Abstract Sentence Classification: Computational Study

**DOI:** 10.2196/54803

**Published:** 2025-03-19

**Authors:** Jiajia Li, Zikai Wang, Longxuan Yu, Hui Liu, Haitao Song

**Affiliations:** 1 Shanghai Artificial Intelligence Research Institute Co., Ltd, Shanghai, China; 2 Xiangfu Laboratory, Jiaxing, China; 3 School of Chemistry and Chemical Engineering, Shanghai Jiao Tong University, Shanghai, China; 4 Inner Mongolia Academy of Science and Technology, Hohhot, China; 5 University of California San Diego, San Diego, CA, United States; 6 Shanghai Civil Aviation College, Shanghai, China

**Keywords:** medical abstract sentence classification, large language models, synthetic datasets, deep learning, Chinese medical, dataset, traditional Chinese medicine, global medical research, algorithm, robustness, efficiency, accuracy

## Abstract

**Background:**

Medical abstract sentence classification is crucial for enhancing medical database searches, literature reviews, and generating new abstracts. However, Chinese medical abstract classification research is hindered by a lack of suitable datasets. Given the vastness of Chinese medical literature and the unique value of traditional Chinese medicine, precise classification of these abstracts is vital for advancing global medical research.

**Objective:**

This study aims to address the data scarcity issue by generating a large volume of labeled Chinese abstract sentences without manual annotation, thereby creating new training datasets. Additionally, we seek to develop more accurate text classification algorithms to improve the precision of Chinese medical abstract classification.

**Methods:**

We developed 3 training datasets (dataset #1, dataset #2, and dataset #3) and a test dataset to evaluate our model. Dataset #1 contains 15,000 abstract sentences translated from the PubMed dataset into Chinese. Datasets #2 and #3, each with 15,000 sentences, were generated using GPT-3.5 from 40,000 Chinese medical abstracts in the CSL database. Dataset #2 used titles and keywords for pseudolabeling, while dataset #3 aligned abstracts with category labels. The test dataset includes 87,000 sentences from 20,000 abstracts. We used SBERT embeddings for deeper semantic analysis and evaluated our model using clustering (SBERT-DocSCAN) and supervised methods (SBERT-MEC). Extensive ablation studies and feature analyses were conducted to validate the model’s effectiveness and robustness.

**Results:**

Our experiments involved training both clustering and supervised models on the 3 datasets, followed by comprehensive evaluation using the test dataset. The outcomes demonstrated that our models outperformed the baseline metrics. Specifically, when trained on dataset #1, the SBERT-DocSCAN model registered an impressive accuracy and *F*_1_-score of 89.85% on the test dataset. Concurrently, the SBERT-MEC algorithm exhibited comparable performance with an accuracy of 89.38% and an identical *F*_1_-score. Training on dataset #2 yielded similarly positive results for the SBERT-DocSCAN model, achieving an accuracy and *F*_1_-score of 89.83%, while the SBERT-MEC algorithm recorded an accuracy of 86.73% and an *F*_1_-score of 86.51%. Notably, training with dataset #3 allowed the SBERT-DocSCAN model to attain the best with an accuracy and *F*_1_-score of 91.30%, whereas the SBERT-MEC algorithm also showed robust performance, obtaining an accuracy of 90.39% and an *F*_1_-score of 90.35%. Ablation analysis highlighted the critical role of integrated features and methodologies in improving classification efficiency.

**Conclusions:**

Our approach addresses the challenge of limited datasets for Chinese medical abstract classification by generating novel datasets. The deployment of SBERT-DocSCAN and SBERT-MEC models significantly enhances the precision of classifying Chinese medical abstracts, even when using synthetic datasets with pseudolabels.

## Introduction

### Background

In the realms of machine learning and artificial intelligence, the significance of data cannot be overstated, yet accessing authentic real-world datasets often presents substantial hurdles, including elevated costs, extended timeframes, and privacy issues. To navigate these obstacles, there is a growing pivot toward the utilization of synthetic datasets. While synthetic datasets have predominantly been associated with computer vision applications, the landscape is changing for natural language processing (NLP) due to advancements in text generation capabilities, largely attributed to the multihead self-attention mechanism integral to the transformer family of models. This trend began with the transformer itself [1] and has since given rise to models such as Bidirectional Encoder Representations from Transformers (BERT) [2], OpenAI GPT [3], Transformer-XL [4], OpenAI GPT-2 [5], and Grover [6]. In recent years, large language models have transformed the field of NLP, demonstrating exceptional performance in a wide range of tasks. These models generate increasingly coherent text, prompting researchers to explore the potential of synthetic datasets. Particularly noteworthy, Zellers et al [6] claimed that their Grover model for conditional text generation outperforms human-generated text in both style and content, especially in the “fake news” and “propaganda” categories, as evaluated by human raters. This highlights the promise of synthetic datasets to improve NLP performance and the continued need to develop text generation techniques.

### Prior Work

#### Sentence-Level Text Classification

Text classification at the sentence level has been explored using various deep learning models, such as convolutional neural networks (CNNs) and recurrent neural networks (RNNs). Kim [7] first proposed a single-layer CNN with pretrained word embeddings for text classification, achieving excellent results. Yang et al [8] developed a 2-level attention mechanism using gate recurrent units for document classification, while Conneau et al [9] introduced a CNN-nonstatic model with character-level CNNs and an average pooling layer. Recently, pretrained models, such as BERT [2], RoBERTa [10], XLNet [11], and ALBERT [12], have also been used for sentence-level text classification tasks. These transformer-based models generate sentence representations and can be combined with various classifiers to achieve state-of-the-art performance. Additionally, hybrid approaches using word-level and character-level CNNs initialized with ELMo [13] or BERT embeddings have been explored to improve the robustness and performance of sentence-level text classification models. Overall, pretrained models have significantly advanced the state-of-the-art in sentence-level text classification, and further research in this area is expected to yield even more sophisticated models.

#### Unsupervised Text Clustering

Unsupervised text clustering is an important task in NLP that groups similar text documents without relying on labeled examples. While traditional methods such as hierarchical agglomerative clustering [14], k-means clustering [15], nonnegative matrix factorization [16], and latent dirichlet allocation [17] have been widely used for this task, recent advances in pretrained embeddings have led to new and competitive methods. These include paragraph vector [18] and USE+KMeans [19], which have shown promising results in various text clustering benchmarks. More recently, BERT-based methods, such as BERT-EMD [20], have been proposed for unsupervised text clustering. Additionally, SBERT [21] and DocSCAN [22] are the most recent methods that generate high-quality text embeddings and use graph-based clustering for unsupervised document clustering. Together, these developments highlight the continued importance of unsupervised text clustering in NLP research, with recent methods based on pretrained embeddings, and SBERT in particular, showing promising results.

#### Synthetic Data

Synthetic data, generated using various techniques such as generative adversarial network (GAN)-based text generation and language model-based data augmentation, has become a popular way to expand small datasets in NLP. These synthetic datasets aim to improve model performance and generalization by providing additional examples for training. For instance, Xu et al [23] used a GAN-based method to generate synthetic data for image captioning, which showed promising results. Similarly, Wang and Wan [24] expanded GAN-based text generation to create synthetic datasets for sentiment analysis, which performed similarly to real data. Additionally, language model–based data augmentation, such as using GPT-2 for auto-completion, has been effective in generating synthetic data for NLP tasks [5]. Zhang et al [25] improved Chinese text classification using a language model–based data augmentation technique, while Zhou et al [26] showed that their language model–based data augmentation method improved low-resource language modeling. However, the use of synthetic data in NLP also has its limitations and potential drawbacks, which require further research. It is important to explore the advantages and disadvantages of synthetic datasets, as well as the different techniques used to generate them, to fully understand their impact on model performance and generalization.

### The Goal of This Study

**Figure 1.**
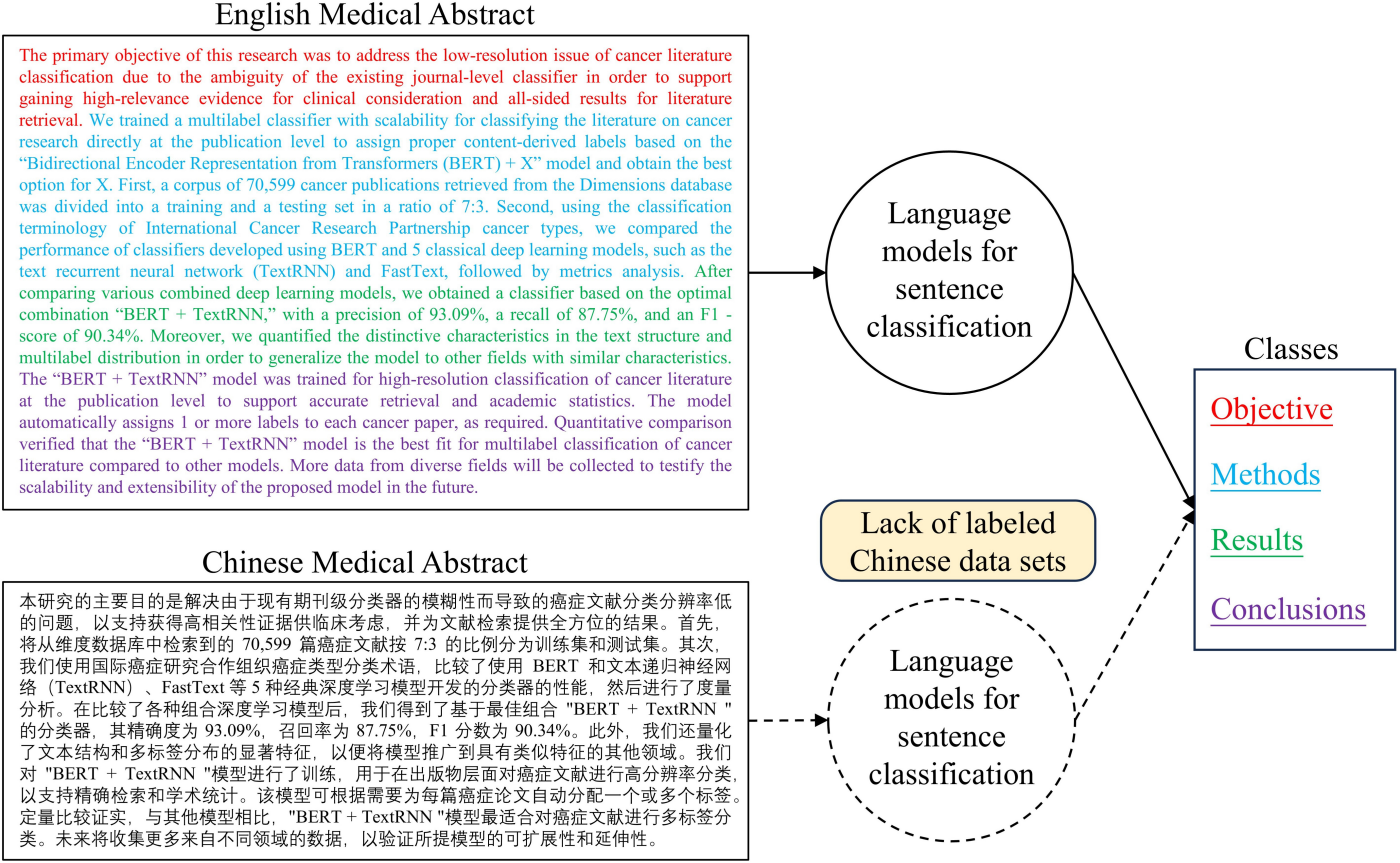
Goal of this study. It has been observed that Chinese medical abstract classification faces a significant number of limitations due to the lack of corresponding labeled datasets. Achieving accurate sentence-level classification in these abstracts will be instrumental in contributing Chinese medical information to the global medical field.

This paper aims to explore the creation and use of synthetic datasets to address the lack of real-world datasets for Chinese medical abstract classification, as there are currently no open-source datasets with sentence-level classification labels for Chinese medical abstracts, as shown in Figure 1. In response to this challenge, we have harnessed the capabilities of GPT-3.5 to generate distinct datasets for the classification of Chinese medical abstracts. Moreover, we designed and trained the clustering and supervised models for this task. Through this innovative approach, this study not only showcases the immense potential of synthetic datasets to bridge the gaps inherent in real-world datasets but also illuminates their profound impact on enhancing the performance of NLP tasks.

## Methods

### Overall Framework

**Figure 2.**
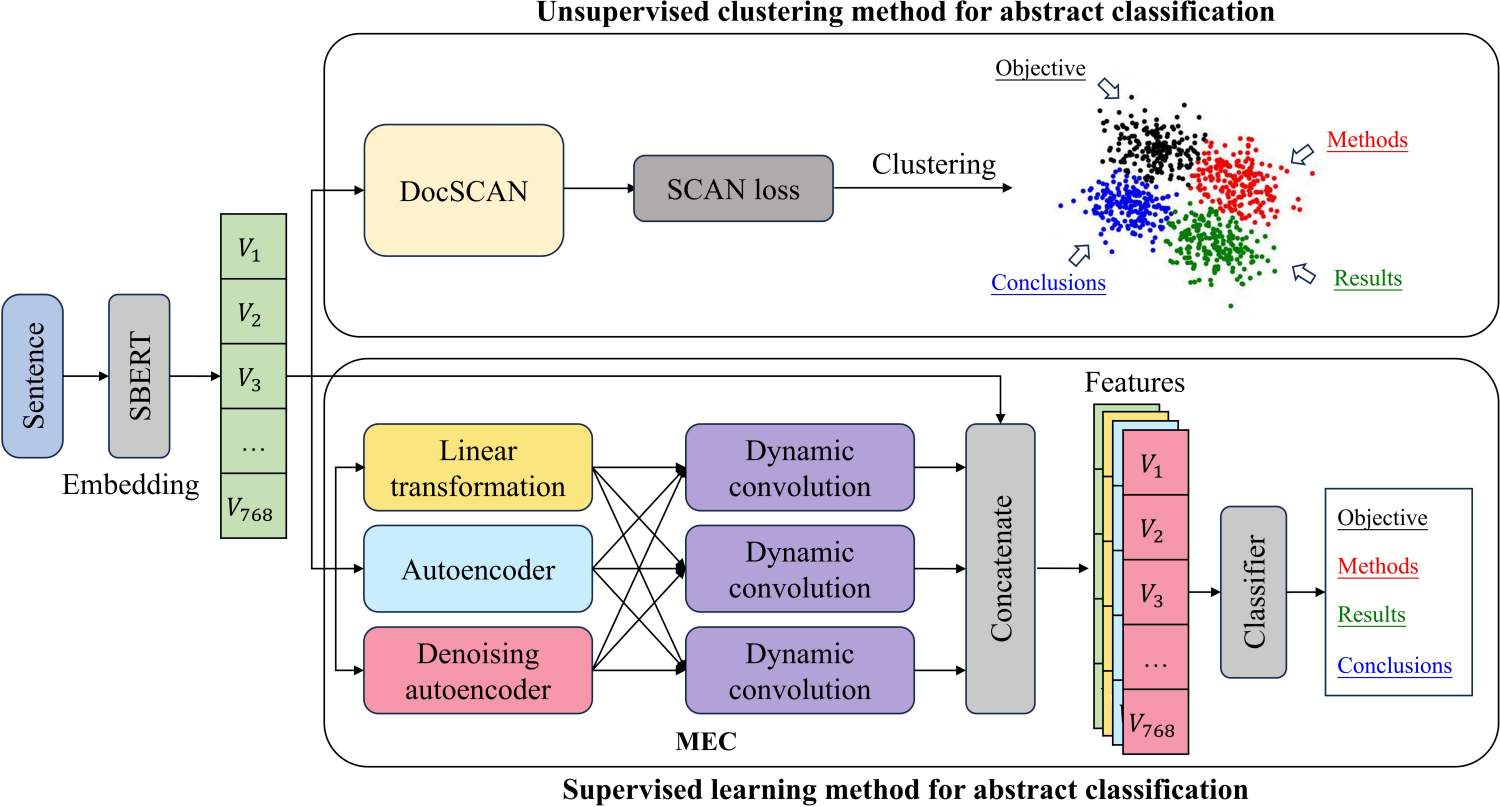
Framework of the proposed methods for abstract classification. The unsupervised clustering method (SBERT-DocSCAN) leverages SBERT embeddings and DocSCAN to group sentences into abstract sections based on clustering. The supervised learning method (SBERT-MEC) uses a multiencoder cascade (MEC) to enhance classification accuracy by extracting and integrating features for abstract sentence classification.

The methodology used in this paper is structured into three distinct sections, systematically addressing the creation and application of synthetic datasets for Chinese medical abstract classification:

1. Synthetic dataset generation: the process begins by translating the PubMed dataset [27] into Chinese and generating 2 distinct synthetic abstract datasets based on provided titles, keywords, disciplines, and categories by GPT-3.5.
2. Unsupervised clustering: this step involves fine-tuning the SBERT model with the synthetic datasets and then using the DocSCAN algorithm to cluster the generated sentence embeddings in an unsupervised manner, termed SBERT-DocSCAN (Figure 2).
3. Supervised classification: a new supervised method, SBERT-MEC, is proposed to classify the synthetic data set, enhancing the ability to accurately categorize synthetic medical abstracts (Figure 2).

### Synthetic Dataset Generation

As there is currently no Chinese dataset for sentence-level classification in the medical abstract field, in this step, we used OpenAI’s text generation model, GPT-3.5, to generate 3 types of small synthetic datasets, which only contain around 15,000 sentences each yet still perform well on classification tasks. The first one is the translated PubMed dataset, which is translated into Chinese from the PubMed 200k RCT dataset by DeepL [28], and we choose 15,000 sentences with clear labels as dataset #1. The second dataset is created by using GPT-3.5 to generate abstracts based on title, keywords, discipline, and category from the CSL dataset [29]. The third dataset is also generated using GPT-3.5, but instead, it is paraphrased with rewritten abstracts assigned pseudolabels as dissimilar to the original text as possible.

**Figure 3.**
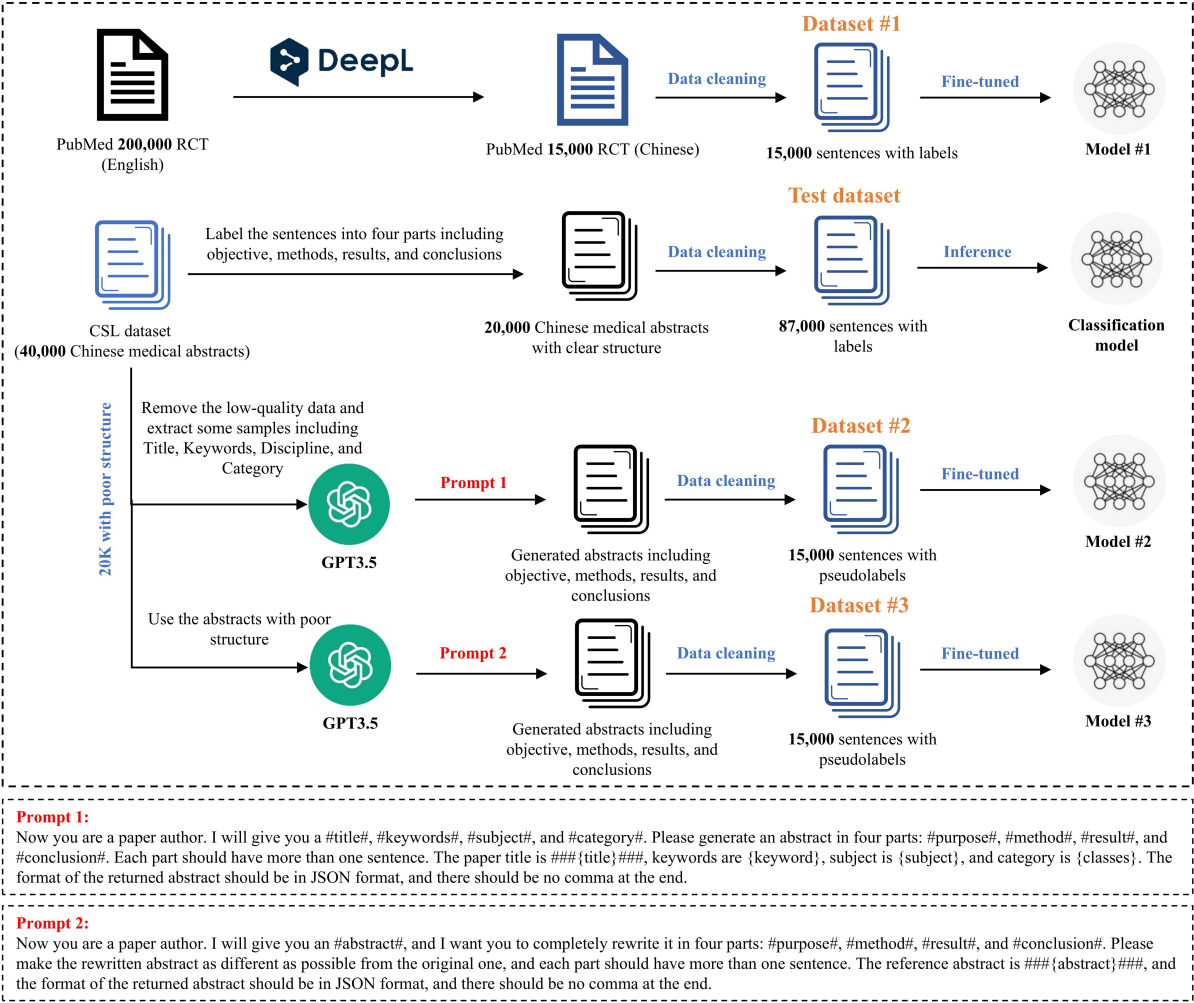
Construction process of the synthetic datasets used for sentence-level classification in the medical abstract field. Dataset #1 is derived by translating the PubMed 200k RCT dataset into Chinese using DeepL and selecting 15,000 sentences with clear labels. Dataset #2 is generated by GPT-3.5, using titles, keywords, disciplines, and categories from the CSL dataset to produce structured abstracts divided into 4 parts (objective, methods, results, and conclusions). Dataset #3 is created by paraphrasing structure-less abstracts using GPT-3.5, generating diverse and rewritten abstracts with pseudolabels. The test dataset comprises 20,000 manually labeled abstracts from the CSL corpus with clear structural divisions. For each of the models in the paper (eg, SBERT), we train them on the training sets separately to produce corresponding ones (eg, SBERT #1, SBERT #2, and SBERT #3).

Generating a diverse training dataset using large language models is a challenge. Even when the generation temperature is set to a high value, these models still tend to generate highly repetitive datasets that lack the diversity required for effective language model training. To address this issue, we selected the CSL corpus as the base corpus, which contains over 40,000 Chinese medical abstracts. Although these abstracts are not annotated at the sentence level, 20,000 of them have clear structure division into the 4 parts mentioned above. We extracted these 20,000 abstracts and manually labeled them as the test dataset. For the remaining 20,000 abstracts without clear structure division, we removed some low-quality data and the corpus of abstracts, then extracted a portion of them as the input corpus for GPT-3.5, which includes corresponding titles, keywords, subjects, and categories. These 4 types of data were inputted into GPT-3.5 to generate abstracts in 4 parts: purpose, method, results, and conclusion. These abstracts were then cleaned to produce a dataset of 15,000 sentences with pseudolabels (dataset #2). Similarly, the structure-less abstracts were inputted into GPT-3.5, which rewrote them to generate clear structure abstracts in 4 parts, resulting in a dataset of 15,000 sentences with pseudolabels (dataset #3). The construction process of the above datasets is shown in Figure 3.

### Unsupervised Clustering

In the second part, we fine-tuned the sentence transformer and then used the DocSCAN algorithm to cluster the synthetic datasets. We chose the sbert-chinese-general-v2 model, which is a model pretrained on the SimCLUE dataset [30], as the base model due to its outstanding performance on embedding Chinese sentences. We fine-tuned the pretrained model on the 3 datasets into 3 models, which is quite crucial as it leads to the overall distribution more inclined toward the functional aspects of each sentence rather than the literal content. We then used these fine-tuned models to embed the input data, followed by clustering by the DocSCAN algorithm which is an algorithm relying on the intuition that a datapoint and its nearest neighbors in representation space often share the same class label, and outperform others on unsupervised classification tasks. Specifically, in this part, we generated embeddings for each sentence in the test set using the 3 fine-tuned sentence transformers in the first part and then used the DocSCAN algorithm to cluster these embeddings for unsupervised abstract classification, which uses the SCAN loss $L_{S}$ to fine-tune the model f(x), defined as:


$$L_{S}=-\frac{1}{\left|D\right|}\sum_{x\in D}\sum_{k\in N_{x}}log\left(f\left(x\right)\cdot f\left(k\right)\right)+\lambda \sum_{i\in C}p_{i}log\left(p_{i}\right)$$

which can be broken down into a consistency loss and an auxiliary loss. The consistency loss aims to ensure that the same label is assigned to a data point and its neighbors. Our model f(⋅) computes a label for a given data point x from the dataset *D*, as well as for each data point *k* in the set of mined neighbors from *x* in $N_{x}$. To achieve this, we calculate the dot product between the output distribution (normalized by softmax(⋅) of datapoint x and its neighbor k. The auxiliary loss is used to achieve regularization via entropy, scaled by a weight λ. This loss encourages the model to distribute probability mass across all clusters C, where $p_{i}$ denotes the assigned probability of cluster i in C by the model. Without this term, there could be a shortcut where all examples collapse into a single cluster. The entropy term ensures that the distribution of class labels resulting from applying DocSCAN tends to be roughly uniform.

### Supervised Classification

In the third part, we develop a supervised learning method, SBERT-MEC, which leverages the proposed multiencoder cascade (MEC) module for feature extraction, designed to enhance the classification of synthetic datasets through refined weak supervision. Figure 2 shows the framework of SBERT-MEC and the modeling process. The first step of the SBERT-MEC is to input the sentence embeddings generated by SBERT into the MEC module, which includes 3 encoders: linear transformation, autoencoder model, and denoising autoencoder model. Formally, given a 768-dimensional sentence embedding V, the linear transformation V transforms the sentence embedding V into W by adding a small random number n:


$$W=L\left(V\right)=\left(V_{1}+n,V_{2}+n,\ldots ,V_{768}+n\right)$$

The autoencoder model can be represented as follows:


$$W={AE}_{\theta }(V)$$

where AE is a fully connected network that maps V into a duplicate embedding W and θ is the parameters of AE. The autoencoder is trained by duplicate pairs from the Quora dataset [31]. The denoising autoencoder (DA), in another way, adds Gaussian noise to the sentence embedding and forms a noisy embedding $V_{N}$, and the training process forces output W to be as close to the original embedding V as possible:


$$V_{N}=V+\epsilon ,\epsilon ∼N\left(0,\sigma^{2}I\right)$$

$$W\;=\;{DA}_{\theta }\left(V_{N}\right)$$

To further enhance performance, 3 embeddings are input into the dynamic convolutional layer for mutual supervision. Specifically, for each embedding, the embeddings generated by the other 2 encoders are concatenated together and used as inputs to supervise this embedding. Finally, 3 output embeddings of the dynamic convolutional layers are concatenated with the original embedding and then put into a classifier to predict test data. This process is repeated for the 3 models fine-tuned in the first part respectively.

To summarize, first, we create synthetic datasets by different methods and leverage them to fine-tune sentence transformers. These datasets are small in size while effective in training classification tasks. Second, fine-tuned models are used on an unsupervised classification algorithm DocSCAN to analyze the performance of these datasets. Further, fine-tuned models are used on a weakly supervised algorithm SBERT-MEC proposed by us, which performs data augmentation on embeddings. This process demonstrates the performances of small synthetic datasets again, as well as the effectiveness of our algorithm.

### Ethical Considerations

This study did not involve human participants, animal subjects, or personally identifiable data. All data used in the research were either synthetic or publicly available, ensuring compliance with ethical guidelines. As such, no ethical approval or informed consent was required. The study adheres to principles of academic integrity and research transparency.

## Results

### Dataset

For empirical evaluation, we crafted 3 datasets: 2 synthetic ones generated using GPT-3.5, and a pre-existing dataset. Each synthetic dataset comprises approximately 15,000 sentences pertinent to medical abstracts. These datasets underwent a meticulous generation process, including cleaning and pseudolabel assignment for sentences within each abstract section. Detailed methodology for creating the synthetic datasets is outlined in the section on Synthetic Dataset Generation. Table 1 illustrates the distribution of sentences across various sections within each dataset.

**Table 1.** Distribution of development datasets. Dataset #1 (translated PubMed dataset with labeled sentences), dataset #2 (GPT-3.5-generated abstracts with pseudolabels), and dataset #3 (paraphrased GPT-3.5-generated abstracts). Each dataset contains 15,000 sentences, divided into 4 sections: objective, methods, results, and conclusion.

Dataset	Classes of sentences				Total
	Objective	Methods	Results	Conclusion	
Dataset #1	3750	3750	3750	3750	15,000
Dataset #2	3750	3750	3750	3750	15,000
Dataset #3	3537	4140	3826	3497	15,000

### Baselines

We selected multiple algorithms of different kinds as baselines to demonstrate the performance of our algorithms. To begin with, we used classical non-BERT models. TextCNN is built upon the CNN paradigm, applying multiple convolutional filters and pooling operations to capture local features of varying lengths within the text. TextRNN [32] leverages RNNs to model the sequential information in the text, capturing dependencies between words. TextRNN-Att [33] extends TextRNN by incorporating attention mechanisms, enabling the model to focus on essential words. TextRCNN [34] combines convolutional and RNNs, simultaneously considering word order and contextual information. DPCNN [35] uses multiple layers of convolution and pooling operations to capture hierarchical text features. FastText [36] is a simple and efficient text classification method based on the bag-of-words model, representing text at the word level.

Additionally, we used BERT-based methods. BERT is a transformer-based pretrained language model, capable of learning rich contextual representations. BERTCNN [37] and BERT-RNN [8] integrate CNNs and RNNs, respectively, on top of BERT, combining the contextual representations from BERT with the ability to extract local features. BERT-RCNN [38] combines BERT with both convolutional and RNNs, simultaneously considering word order and contextual information. BERT-DPCNN [39] incorporates a deep pyramid CNN on top of BERT, combining the contextual representations from BERT with multilevel feature extraction capabilities. ERNIE [40] is another transformer-based pretrained language model, built upon BERT with further improvements and optimizations, with better performance in Chinese contexts.

In our ablation study, we conducted an experiment using the k-means algorithm to cluster SBERT embeddings for comparative analysis with DocSCAN. Additionally, we examined the impact of omitting the dynamic convolutional layer in the MEC module. Subsequent experiments involving BERT serve as an extension of this analysis, further exploring the effects of removing 3 encoders within MEC.

### Experiment Settings

In this study, we used the PyTorch framework and Python for all experiments. Data processing and model training were performed on an NVIDIA GeForce RTX 3090 (24G) GPU and an Intel(R) Core(TM) i9-10900K CPU @3.70GHz. Experimental parameters are detailed in Table 2. To mitigate overfitting, a dropout rate of 0.5 was used, randomly deactivating 50% of neuron connections during training. We conducted training over 30 epochs using either the Adam or BertAdam optimization algorithms, with a learning rate set at 5e-5. Training batches comprised 64 samples each, and the RNN featured a hidden size of 256. The CNN component used filter sizes of (2, 3, 4), with a total of 256 filters. Input sequence length was capped at 64 tokens, and the feature dimension was maintained at 768. Pretrained models used in the experiments included BERT (bert-base-chinese), ERNIE (ernie,-3.0-base-zh), and SBERT (sbert-chinese-general-v2), which were critical to the study’s execution and result analysis.

**Table 2.** Settings for training models.

Parameter	Setting
Dropout	0.5
Epoch number	30
Optimizer	Adam/BertAdam
Learning rate	$e^{-5}$
Batch size	64
RNN^a^ hidden size	[2]
CNN^b^ filter number	256
Max length	64
Feature dimension	768
BERT^c^	Bert-base-chinese
ERNIE	Ernie-3.0-base-zh
SBERT	Sbert-chinese-general-v2

a RNN: recurrent neural network.

b CNN: convolutional neural network.

c BERT: Bidirectional Encoder Representations from Transformers.

### Evaluation Metrics

The experiments used accuracy and *F*_1_-score to evaluate the model performance. The formulas for each index are as follows:


$$Accuracy=\;\frac{TP+TN}{TP+TN+FP+FN}$$

where TP (true positive) corresponds to the number of instances correctly predicted as positive; TN (true negative) corresponds to the number of instances correctly predicted as negative; FP (false positive) corresponds to the number of instances incorrectly predicted as positive; FN (false negative) corresponds to the number of instances incorrectly predicted as negative.


$$Precision=\;\frac{TP}{TP+FP}$$

$$Recall=\;\frac{TP}{TP+FN}$$

$$F_{1}\text{-}score=\;\frac{2\times Precision\times Recall}{Precision+Recall}$$

In the above formulations, the Precision measures the proportion of correctly predicted positive instances out of the total instances predicted as positive and Recall measures the proportion of correctly predicted positive instances out of the total actual positive instances. $F_{1}\text{-}score$ is the harmonic mean of precision and recall, providing a single value that considers both metrics. This technique is highly effective for imbalanced datasets, where disparities in class sizes can skew model performance. It helps ensure fair representation of all classes, enhancing model reliability.

### Model Performance

Table 3 shows the performance of various algorithms across different training datasets when evaluated on the test dataset. Notably, when trained on dataset #1, the SBERT-DocSCAN algorithm emerges as the leading performer, securing an accuracy and *F*_1_-score of 0.8985 on the test dataset. This standout performance highlights the algorithm’s capability to classify medical domain data with high precision. Additionally, the SBERT-MEC algorithm also displays comparable performance on the same dataset, with an accuracy and *F*_1_-score of 0.8938, making it the second most effective algorithm in our evaluation. For Data se t#2, the SBERT-DocSCAN and SBERT-MEC algorithms again demonstrate superior performance. SBERT-DocSCAN leads with exceptional accuracy and *F*_1_-score of 0.8983, reinforcing its effectiveness in managing generated data. Meanwhile, SBERT-MEC remains a strong contender with an accuracy and *F*_1_-score of 0.8673, marking it as the second most proficient algorithm for this dataset. Furthermore, considering dataset #3, SBERT-DocSCAN and SBERT-MEC algorithms consistently demonstrate outstanding performance. SBERT-DocSCAN achieves the highest accuracy and *F*_1_-score of 0.9130, affirming its effectiveness in handling paraphrased data. Similarly, the SBERT-MEC algorithm achieves the second-highest accuracy and *F*_1_-score of 0.9039 and 0.9035, respectively, highlighting its competence in dealing with paraphrased data. Additionally, Table 4 illustrates the confusion matrices of the SBERT-DocSCAN model on the test dataset using different training datasets.

In summary, the comprehensive analysis of the table elucidates that the SBERT-DocSCAN and SBERT-MEC algorithms consistently outperform other algorithms across multiple datasets. The superior performance of these algorithms in terms of accuracy and F1 scores underscores their significance and efficacy in the domain of text classification.

**Table 3.** Comparative performance of various models on the test dataset^a^.

Method	Training on dataset #1		Training on dataset #2		Training on dataset #3	
	Accuracy	*F*_1_-score	Accuracy	*F*_1_-score	Accuracy	*F*_1_-score
TextCNN	0.6122	0.6074	0.7843	0.7795	0.6789	0.6755
TextRNN	0.5045	0.5413	0.7315	0.7321	0.7295	0.7239
TextRNN-Att	0.6439	0.6421	0.7114	0.6955	0.7521	0.7521
TextRCNN	0.6562	0.6543	0.7642	0.7643	0.7439	0.7392
DPCNN	0.6953	0.6967	0.6947	0.6988	0.6758	0.6759
FastText	0.6562	0.6543	0.7208	0.7101	0.7391	0.7371
BERT^b^	0.7842	0.7857	0.8426	0.8400	0.7348	0.7083
BERT-CNN	0.7338	0.7340	0.8242	0.8213	0.7181	0.7136
BERT-RNN	0.8548	0.8546	0.8536	0.8532	0.7955	0.7882
BERT-RCNN	0.8245	0.8253	0.8313	0.8252	0.8364	0.8332
BERT-DPCNN	0.7839	0.7850	0.8186	0.8186	0.8301	0.8304
ERNIE	0.8801	0.8808	0.8681	0.8675	0.8895	0.8882
SBERT-Kmeans	0.8875	0.8875	0.8709	0.8709	0.8788	0.8788
SBERT-DocSCAN	0.8985	0.8985	0.8983	0.8983	0.9130	0.9130
SBERT-MEC	0.8938	0.8938	0.8673	0.8651	0.9039	0.9035

a It includes traditional models, BERT-based improved models, and the model proposed in this paper. These models were trained on 3 distinct datasets and subsequently evaluated on the test dataset in terms of accuracy and *F*_1_-score.

b BERT: Bidirectional Encoder Representations from Transformers.

**Table 4.** Confusion matrices for the SBERT-DocSCAN model on the test dataset^a^.

Label or cluster	0	1	2	3	Total	Recall
Model training on dataset #1						
Objective	20,302	1304	25	490	22,051	0.9175
Methods	1533	19,825	448	137	21,943	0.9035
Results	179	901	18,676	2075	21,831	0.8555
Conclusions	649	280	861	19,914	21,704	0.9175
Total	22,593	22,310	20,010	22,616	78,647^b^	0.8985
Precision	0.8595	0.8886	0.9333	0.8805	0.8985	—^c^
Model training on dataset #2						
Objective	20,250	1247	24	530	22,051	0.9183
Methods	705	20,496	397	345	21,943	0.934
Results	210	626	18,005	2990	21,831	0.8247
Conclusions	102	141	1585	19,876	21,704	0.8983
Total	21,267	22,510	20,011	23,741	78,647^b^	0.8983
Precision	0.9522	0.9105	0.8998	0.8372	0.8983	—
Model training on dataset #3						
Objective	20,401	251	129	1447	22,051	0.9178
Methods	505	19,673	1325	29	21,943	0.9137
Results	288	1655	19,864	593	21,831	0.8868
Conclusions	749	252	386	19,982	21,704	0.9131
Total	21,943	21,831	21,704	22,051	78,647^b^	0.913
Precision	0.9297	0.9012	0.9152	0.9062	0.913	—

a This table displays the confusion matrices for the SBERT-DocSCAN model, which was trained separately on 3 distinct training datasets. The matrices detail the accuracy and recall for each category when evaluated on the test dataset.

b Out of 87,529.

c Not applicable.

### Ablation Study

To assess the impact of various submodules, we conducted ablation studies. We began by training 2 clustering models with identical structures (SBERT-DocSCAN). The key distinction between them was that 1 model was fine-tuned on our datasets, while the other was not. In addition, we trained 3 supervised models on different datasets and evaluated their performance on the test dataset. Notably, 1 of these models was the SBERT-MEC, which lacked the proposed dynamic convolution (DC) module. The results, presented in Table 5, clearly demonstrate that the SBERT-DocSCAN method, when fine-tuned with SBERT, outperforms others in terms of efficiency. Furthermore, within the supervised learning category, the SBERT-MEC model equipped with the DC module surpassed those lacking this module, underlining the value of the DC module in enhancing model performance.

**Table 5.** Performance of ablation model on test dataset^a^.

Method	Training on dataset #1		Training on dataset #2		Training on dataset #3	
	Accuracy	*F*_1_-score	Accuracy	*F*_1_-score	Accuracy	*F*_1_-score
SBERT-DocSCAN (without finetune)	0.3891	0.3895	0.3891	0.3895	0.3891	0.3895
SBERT-DocSCAN (with finetune)	0.8985	0.8985	0.8983	0.8983	0.9130	0.9130
BERT^b^	0.8330	0.8324	0.8424	0.8421	0.8406	0.8397
SBERT-MEC^c^ (without DC)^d^	0.8714	0.8717	0.8390	0.8332	0.8736	0.8735
SBERT-MEC (with DC)	0.8938	0.8938	0.8673	0.8651	0.9039	0.9035

a This table compares the performance of 2 SBERT-DocSCAN clustering models—one fine-tuned on our datasets and the other not—and 3 supervised models, including an SBERT-MEC model without the dynamic convolution module. All models were evaluated on the test dataset.

b BERT: Bidirectional Encoder Representations from Transformers.

c MEC: multiencoder cascade.

d DC: dynamic convolution.

## Discussion

### Principal Findings

This study addresses the critical lack of real-world datasets for Chinese medical abstract classification by leveraging GPT-3.5 to generate synthetic datasets and developing models tailored for this task. Our findings confirm that synthetic datasets, when carefully designed, can match or surpass the performance of manually labeled datasets in sentence-level classification tasks. The SBERT-DocSCAN and SBERT-MEC models developed in this study demonstrate the potential of clustering and supervised approaches to effectively classify Chinese medical abstracts, illustrating the profound impact of synthetic datasets on enhancing NLP tasks.

Classical non-BERT models, such as TextCNN [7], TextRNN [32], and TextRCNN [34], exhibit strong performance in capturing local or sequential features. However, these models lack the deep contextual understanding provided by transformer-based models. While FastText [36] offers a lightweight and efficient alternative, its bag-of-words approach limits its ability to capture complex semantic relationships. These limitations underscore the advantages of leveraging pretrained language models in tasks that demand rich contextual understanding.

BERT-based models significantly improve performance by providing deep contextual representations. The success of models like BERT-CNN [37] and BERT-RCNN [38] aligns with prior studies, which highlight the effectiveness of combining BERT embeddings with convolutional and recurrent structures for enhanced local and sequential feature extraction. However, our proposed SBERT-DocSCAN and SBERT-MEC models outperform these baselines, indicating the added value of advanced clustering methods and DC layers. Specifically, DocSCAN [22], with its graph-based clustering approach, demonstrates superior clustering quality compared to k-means.

The ablation study further emphasizes the contributions of individual components in our models. The removal of the dynamic convolutional layer in the MEC module resulted in a notable decline in performance, highlighting its role in refining language representations. Similarly, the omission of the 3 encoders within MEC led to a significant reduction in accuracy, underscoring the importance of multiencoder architecture in capturing diverse linguistic features. These findings align with the broader NLP literature [39], which emphasizes the benefits of combining multiple feature extraction techniques for enhanced model performance.

Our exploration of ERNie [40], a BERT-based model optimized for Chinese contexts, further validates the importance of leveraging models tailored to specific languages and domains. While ERNie offers improvements over standard BERT in Chinese text classification, it does not surpass the performance of our SBERT-based approaches. This suggests that task-specific architectural innovations, such as the integration of clustering methods and DC modules, can provide greater benefits than domain-specific pretraining alone.

Overall, these comparisons and ablation studies highlight the robustness and versatility of our proposed methods, providing valuable insights into the design of models for sentence-level text classification tasks. By demonstrating the effectiveness of integrating advanced clustering methods, DC layers, and multiencoder architectures, this study contributes to the growing body of research focused on optimizing transformer-based models for real-world applications.

### Limitations

While promising, our proposed methods have 2 main limitations. First, the quality of synthetic data heavily relies on the GPT-3.5 model, with performance contingent on effective prompt design. Refining prompt engineering strategies will be essential for future improvements. Second, the 2-stage approach used in both models results in an increased parameter size, raising concerns about computational resource efficiency. Further optimization is necessary to address these scalability challenges, especially in real-world applications where resources may be limited.

### Conclusions

This study demonstrates the significant potential of synthetic datasets, generated using GPT-3.5, in addressing the scarcity of labeled datasets for Chinese medical abstract classification. Our findings reveal that compact synthetic datasets can achieve performance comparable to, and in some cases surpass, that of manually labeled datasets. The proposed SBERT-DocSCAN and SBERT-MEC models further highlight the benefits of combining advanced clustering techniques, multiencoder architectures, and DC modules, showcasing their ability to enhance sentence-level classification tasks in specialized domains. These contributions provide valuable insights for leveraging generative artificial intelligence in NLP applications.

Beyond this specific task, this work underscores the transformative potential of synthetic datasets in reducing reliance on costly manual labeling, enabling broader adoption of NLP technologies in resource-limited fields. Future research can expand upon these findings by exploring more sophisticated data generation strategies, optimizing model architectures for efficiency, and fostering interdisciplinary collaborations to develop tailored solutions for complex, real-world challenges. By bridging gaps in data availability, this study provides a foundation for advancing NLP capabilities in medical and other specialized domains.

## Abbreviations

BERT: Bidirectional Encoder Representations from Transformers
CNN: convolutional neural network
DC: dynamic convolution
GAN: generative adversarial network
MEC: multiencoder cascade
NLP: natural language processing
RNN: recurrent neural network
